# Prospective Changes in Health-Related Quality of Life and Emotional Outcomes in Kidney Transplantation over 6 Years

**DOI:** 10.1155/2011/671571

**Published:** 2011-07-21

**Authors:** Konstadina Griva, Jan Stygall, Juan Hui Ng, Andrew Davenport, Mike J. Harrison, Stanton Newman

**Affiliations:** ^1^Department of Psychology, National University of Singapore, 9Arts Link AS402/28, Singapore; ^2^Unit of Behavioural Medicine, University College London, Riding House Street, London W1 8AA, UK; ^3^Health Services Research Group, City University, London, College Building Room A224, St John Street, London EC1V 0HB, UK; ^4^Department of Nephrology, University College, London, and Royal Free Hospital, Pond Street, London NW3 2QG, UK

## Abstract

Little is known on long-term outcomes in kidney transplantation. This study evaluated changes and predictors of generic and transplantation-specific health-related quality of life (HQoL) over six years in *N* = 102 kidney transplant survivors using the Short-form Health Survey-36 and the Transplant Effects questionnaire. Mixed models analysis was used to determine long-term outcomes. Emotional HQoL improved over time: Mental Component score, Mental Health, Energy (*P*s = .000). Physical HQoL deteriorated: Physical Component Score (*P* = .001), Pain (*P* = .002). LRD transplant recipients had greater decline in physical functioning (*P* = .003) and PCS (*P* = .000) compared to cadaver recipients. Worry about the transplant (*P* = .036) and feelings of responsibility (*P* = .008) increased significantly over time. Worry about the transplant and perceived ability to work predicted 12.7% and 31.1% in variance in MCS and PCS, respectively. Efforts should be made to maintain HQoL and emotional outcomes with ongoing monitoring and support programs throughout the course of posttransplant care.

## 1. Introduction

Kidney transplantation is the treatment of choice for selected patients with end-stage renal disease (ESRD). Health-related quality of life (HQoL) has emerged as an important outcome to quantify the success of transplantation in the long term [1, 2]. Although HQoL has been systematically assessed to uniformly document a positive effect of transplantation (with larger gains in the dimensions of HQoL most affected by physical health and more modest improvements in areas affected by psychosocial functioning), there is increasing recognition that transplantation generates unique demands and challenges for patients, and families alike [3–5]. 

The generic HQoL questionnaires provide useful and important information on the multiple dimensions HQoL such as functional status, pain, mood and social welfare, yet they fail to capture issues that are unique to transplant patients [6]. Issues related to the donation, feelings of guilt, concerns about viability of graft and uncertainty about the future have been shown to define transplantation experience [7–11] and are hence fundamental to understanding overall sense of well-being in transplant recipients. This has led to the development and use of the Transplant Effects Questionnaire, a validated condition-specific questionnaire for the assessment of organ transplant recipients [6, 7, 12, 13].

As graft survival has improved dramatically in last decades [14] the question of long-term outcomes in kidney transplantation becomes increasingly important. The preponderance of literature in patient-reported outcomes in kidney transplantation to date has focused on early post operative period [15] or cross-sectional comparisons of recipients [16] and/or long-term transplant survivors with their dialysis counterparts [17]. Significant associations are typically found between time elapsed since transplantation and symptom experience/burden which may compromise adjustment and HQoL. Data on longitudinal changes over the posttransplantation period are sparse and typically do not extend to more than 2 years posttransplant [18–20]. Our previous study comparing livings related donor (LRD) and cadaver tran-plant recipients [7], like many on patient-reported outcomes in kidney transplantation was cross sectional which limits conclusions as to the trajectory and course of transplantation-specific outcomes between the two transplant groups. Outcomes focusing on longitudinal changes in HQoL and Transplantation specific outcomes are largely lacking in the literature [21].

There is need for further prospective studies using generic and transplantation specific instruments to document long-term patient outcomes beyond the immediate and short term post- transplantation period. There is also a need to establish predictors of long-term outcomes in this population. 

The current study examines 6-year changes in HQoL and transplantation-specific outcomes in a cohort of kidney transplant recipients. It also compares reported outcomes in living related donor and cadaver transplant recipients and explores whether demographics and clinical variables are predictors of long-term transplantation outcomes.

## 2. Materials and Methods

### 2.1. Design

This is a prospective study in which a baseline kidney transplant cohort was reassessed after a 6 years followup period using the same instruments. (Participants were only followed up until 6-year from baseline assessment due to the planned relocation and merging of one of the transplant units with another renal unit. Thus, it became difficult, if not impossible, to follow patients' health outcomes.)

### 2.2. Participants

This prospective study evaluated patient-reported outcomes at two points in time-on an average of 6 years apart. Kidney transplant recipients from two transplant centres (Middlesex Hospital and Royal Free Hospital, London, UK) who participated in a questionnaire study of HQoL and emotional responses between October 1999 and February 2002 were invited to the followup study. The study protocol was approved by the Institutional Review Board and informed consent was obtained from each participant. Clinical practice and health care procedures (post-transplantation followup care and patient support services) were identical on both hospital sites. There were no known changes at organizational and health care system structures during study window. 

Target study subjects were the subgroup of kidney transplant patients from our previous cross-sectional study [7] who still had functioning grafts. Multiorgan recipients and recipients under 18 years of age at the time of evaluation, patients who had an acute rejection or infection within a month of data collection, had dementia, mental illness, or severe visual/hearing impairment were excluded. Data on patients who died or lost their graft and were placed back on dialysis are also not reported in this paper.

### 2.3. Procedure

Eligible participants were approached by letter or by the Clinical Nurse Specialist to participate in the 6-year followup study. Following consent into the study, participants were requested to undertake a single assessment conducted by a trained psychologist. Assessment sessions were scheduled at patients' convenience with most assessments coinciding with one of their regular check-up outpatient appointments.

### 2.4. Measures

Sociodemographic and medical information were collected at both time points. These included information on age, gender, education level, relationship status (married/cohabitating relationship or single, separated, widowed), occupation (full/part time or unemployed, retired or looking after home/family) and financial situation (annual income; change in income categorized as up, down, or stable). Perceived ability to work was assessed by a single question developed by Evans et al. [22]: “are you now able to work full time, part time, or not at all.”

Medical and transplant-related data were extracted from medical records: primary kidney disease diagnosis, medications (including current immunosuppressive regime), vintage (time elapsed since renal replacement; time elapsed since transplant), donor type (cadaver versus living related), relationship to donor number of infection and rejection episodes, comorbidities. The presence/absence of seven of the most common comorbid disorders (plus an “other” category) was recorded. These included diabetes mellitus, hypertension, ischaemic heart disease, peripheral vascular disease, cancer, bone diseases and chronic obstructive airways disease. All these socioeconomic and clinical parameters were considered both in terms of baseline levels and change over the followup period. For each variable a change score was calculated comparing the data at followup with that at baseline. Changes in these clinical variables were defined as improving, static, or worsening with the static group being treated as the reference group. 

Charlson Comorbidity Index (CCI) was also computed to quantify comorbid illness. CCI scores were calculated using the method previously described by Beddhu et al. [23].

Laboratory data were also recorded (eGFR). Glomerular filtration rate (GFR) was measured following the intravenous administration of 3 MBq 51Cr-EDTA diluted to 10 mL on 0.1% w/v excess EDTA solution [24].

Transplantation-specific emotional and behavioural outcomes were measured using the Transplant Effects Questionnaire (TxEQ) [6]. The 23-item TxEQ contains fve sub-scales that assess worry about the transplant; feelings of guilt towards the donor, disclosure of transplantation medication adherence and perceived responsibility to do well. Subscale scores are expressed as a mean by dividing the total score by the number of items, hence ranging from 1 to 5. Higher scores signify more worry about the transplant, more guilt, more disclosure, more perceived responsibility, respectively, and greater adherence. The questionnaire has data to support its internal structure and factorial validity and has been found to have acceptable internal consistency, test–retest reliability and face validity [6].

HQoL was measured with the 36-item Medical Outcome Study Short Form Health Survey (SF-36) [25]. The UK version 2 of the SF-36 was used to ensure face validity and maximize acceptability in British participants [26]. The SF-36 is a generic multidimensional measure of HQoL that contains eight sub-scales representing physical functioning (PF), social functioning (SF), role limitations due to physical health problems (RPh), role limitations due to emotional problems (REm), mental health (MH), vitality (VT), bodily pain (BP) and general health perceptions (GH). Sub-scales scores range from 0–100 with higher scores indicating better HQoL. In addition, summary scores were calculated for physical (PCS) and mental (MCS) components [27]. To facilitate interpretation and comparisons to the norms, normative-based scoring was used [27]. Normative-based scoring involves a linear *t*-transformation to ensure that all SF-36 sub-scales and summery scores had a mean of 50 and a SD of 10 in the general UK population [28]. The SF-36 has been proved reliable and valid in various demographic and patient populations including ESRD and transplant patients [29, 30]. A difference of five-points in a particular dimension is considered a minimal clinically significant change [31].

Patients' levels of functional ability to perform activities of daily life was determined by the Karnofsky performance status [32] completed by Transplant nurses. Scores range from 0–100 with 0 indicating death and 100 indicating full capacity to perform normal activity. In general scores 50–70 represents an individual who requires additional assistance and inability to work and a score lower than 50 represents needs for hospitalization, nursing care or institutionalization [32–34].

### 2.5. Statistical Analysis

Data analyses were performed using Statistical Package for Social Sciences statistical software for windows (Version 17.0; SPSS Inc., Chicago, IL). Descriptive statistics were performed as appropriate, depending on measurement level and distribution of the data for the sample population and the subgroups. Analyses of variance were performed to test between-group differences, and general linear models for repeated measures assessed intra-individual effects for the study cohort. Trajectories of change (improvement; deterioration; stable) were also calculated to examine change in HQoL in individual participants. Classification was based half SD (5 points) to determine significant change [31, 35].

Inferential statistics included Pearson's or Spearman's correlations (as appropriate), cross tabulations, chi-square, or Fischer's exact test to investigate the impact of relevant variables (absolute baseline and change scores) on HQoL. Hierarchical multiple regression analyses were used to analyze predictive HQoL variables. Variables were entered into the regression model when a significant relationship using bivariate analyses was detected. Baseline levels were included as predictors under forced entry in first step. The level of significance was set at *P* less than  .05.

## 3. Results

### 3.1. Study Population

A total of 102 out of 172 (59.3%) eligible transplant survivors from the original cohort [7] participated in the six-year followup assessment at a mean interval of 76.48 (7.28) months since baseline assessment. Transplant vintage, that is, time elapsed since transplantation at study entry/baseline, was 8.08 (SD = 6.89) years.

Nineteen patients (*N* = 19) refused to participate because of frail/poor health, *N* = 16 due to time commitments, *N* = 16 due to burden of work involved or disinterest; *N* = 6 were undecided, and *n* = 2 were unable to be reached in listed contact numbers/addresses or at their scheduled hospital appointments during the study window. 

Patient survival was 87.8%, and transplant survival was 85.3% in the study period. Mortality was due to cardiovascular reason/events (*N* = 27), malignancy (*N* = 7), and other causes (*N* = 5). Cumulative 6-year rates of infection episodes were  .08 (1.2) per patient. A total of *N* = 38 of patients who lost their transplant were treated with hemodialysis, *N* = 13 were on peritoneal dialysis regimes and *N* = 11 patients were attending low clearance clinic (Stage 3–5 chronic kidney disease).

**Figure 1:**
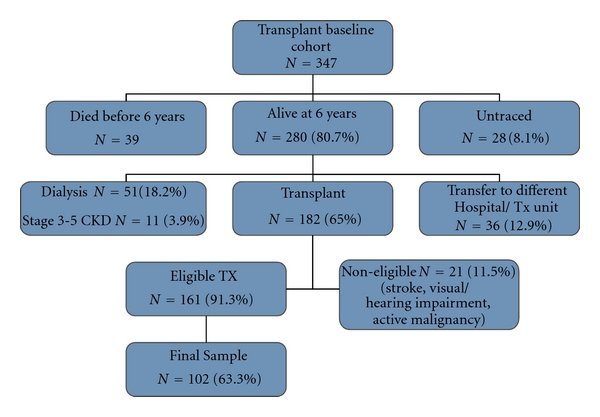
Study cohort.

The paper reports findings on the transplant survivors (see Figure 1 for information of the cohort). The mean age of this group was 46.57(14.21) years; 58.8% of patients were males and most were cadaver transplant recipients (79.4%). 

Immunosuppressive medications were cyclosporine (63.7%) or tacrolimus (32.3%), mycophenolate mofetil (7.8%) and/or azathioprine, 34.3%), and steroids reflecting the state of the art immunosuppression when these patients were transplanted. 

Demographic and medical information showed few differences between responders and nonresponders. Non responders had lower Karnofsky score (*P* = .042) and reported more comorbid conditions (*P* = .023), albeit there were no differences in Charlson Comorbidity Index scores or comorbidities diagnoses abstracted through medical records. All other socioeconomic parameters (e.g., gender, income, work, and relationship status) and clinical markers (e.g., GFR, source of transplant, total ESRD time or transplant vintage, rejection episodes, hospitalisation days) were comparable between responders and non responders. Table 1 details the baseline demographic and clinical characteristics of the study sample and two transplant subgroups.

Socioeconomic and clinical parameters remained largely unchanged across assessments. Relationship status for *N* = 85 (83.3%) patients was unchanged across the two assessments, *N* = 9 (8.8%) respondents reported having lost their spouse/partner through divorce or widowing and *N* = 4 (3.9%) reported change from single/divorcee status to getting married or being in a cohabiting relationship at the 6-year followup.

A total of 19 patients reported changes in employment status with *N* = 12 (12.2%) patients who were employed at baseline, no longer being employed at followup and *N* = 7 (7.1%) patients who were not employed at baseline resuming work. The vast majority had no change in occupational details (*N* = 74; 80.4%). 

Perceived ability to work remained unchanged in *N* = 75 (76%), *N* = 6 (6%) respondents downgraded ability to work and *N* = 18 (18%) reporting improvements in ability to work from baseline to 6-year followup. 

Likewise income was undifferentiated in vast majority of respondents (*N* = 61 (89.2%) of respondents), *N* = 1 (1.5%) reported lower income and *N* = 6 (9.2%) reported higher income at followup compared to baseline.

Rates of comorbid conditions (i.e., diabetes mellitus, hypertension, ischemic heart disease, peripheral vascular disease, cancer, bone diseases and chronic obstructive airways disease) were comparable across assessments; mean GFR levels were significantly lower at 6-year followup reflecting process of chronic rejection (mean GFR at followup = 35.5, SD = 16.8; *F* = 3.42, *P* = .04).

### 3.2. Patient Outcomes: HQoL and TXEQ

The observed SF-36 scores across assessments were comparable to those in general population (within 1 SD of norms; normative mean = 50; SD = 10) with the exception of the physical functioning and general health perception scores which were slightly lower than 1 SD below population norms.

Our first research question was whether patient reported outcomes changed over time.

Repeated measures ANOVAs revealed significant differences in 6 of 10 SF-36 scores (See Table 2). Physical HQoL declined from baseline to 6-year followup: physical component score (*F* = 14.87, *P* = .001), Pain (*F* = 10.04, *P* = .002). Emotional HQoL indicators on the other hand significantly improved over time: mental component score (*F* = 16.64,   *P* = .000) mental health (*F* = 41.49, *P* = .000), vitality (*F* = 13.54, *P* = .000); the only exception being role-emotional which was worse at followup assessment (*F* = 4.49; *P* = .037). 

Significant differences over time were found for two of TXEQ subscales with worry about graft viability (*F* = 4.52, *P* = .036) and perceived responsibility to do well increasing over time (*F* = 7.34, *P* = .008). 

As time since transplantation was varied, analyses were repeated using transplant vintage at study entry (baseline) as a between-subject factor to compare outcomes in patients who had their graft for less than 2 years (19.6%; *N* = 20) with those who had their grafts between 2–5 years (22.5% (*N* = 23) and those with more than 5 years post-transplant 57.8% (*N* = 59). Results replicated the same pattern of changes in SF-36 and TxEQ scores in all subgroups with the exception that there were no significant changes across time in role limitations due to emotional problems (*P* = .18). There were no significant group or interaction effects in any of the outcomes suggesting that transplant vintage had no effect on the study outcomes across time. 

#### 3.2.1. Patterns of Intraindividual Change in HQoL

To evaluate patterns of intra-individual change the two summary HQoL scores (physical component score; mental component score) and frequencies of patterns were analyzed for the slope between time points (from baseline to 6 years): worse (scores at followup ≤ 5 points, i.e., 0.5 SD than baseline), improved (scores at followup ≥ 5 points, i.e., 0.5 SD than baseline), stable (scores at followup within ±4.99 points of baseline). The 0.5 SD was used as this represents the minimally clinical significant difference [31, 33]. 

When evaluating the breakdown of participants into the 3 patterns with respect to physical component score, *N* = 23 (30.7%) had no change in physical component score, *N* = 40 (53.3%) worsened, and *N* = 12 (16.0%) improved over time. 

Similar analysis on mental component score scores revealed that *N* = 34 (45.3%) had no change in mental component score, *N* = 25 (33.3%) worsened, and *N* = 16 (21.3%) improved. 

ANOVAs followed by post hoc comparisons indicated that patients who reported improvements on physical component score scores have had their grafts for longer (mean = 11.01; SD = 2.46) compared to the no change physical component score group (mean = 4.92, SD = 4.71) (*F* = 7.34, *P* = .011). 

The only variable to differentiate between the mental component score groups was TXEQ worry change scores. Post hoc comparisons indicated that patients whose emotional HQoL (i.e., mental component score) worsened experienced increased worry about the viability of their graft (mean worry change score = 2.18, SD = 4.92) compared to patients whose mental component score improved (mean worry change score = −2.50, SD = 7.39) (*F* = 5.35, *P* = .027).

### 3.3. Outcomes across Time between Cadaver and Living Related Transplant Recipients

Comparisons of outcomes by transplant source showed that HQoL levels were comparable for the two transplant groups with the exception of physical functioning and physical component scores.

Repeated measures ANCOVAs controlling for casemix differences (i.e., age, income and duration of transplant) indicated significant interaction effects in physical functioning (*F* = 7.07; *P* = .009) and physical component score (*F* = 6.03, *P* = .017). 

**Figure 2:**
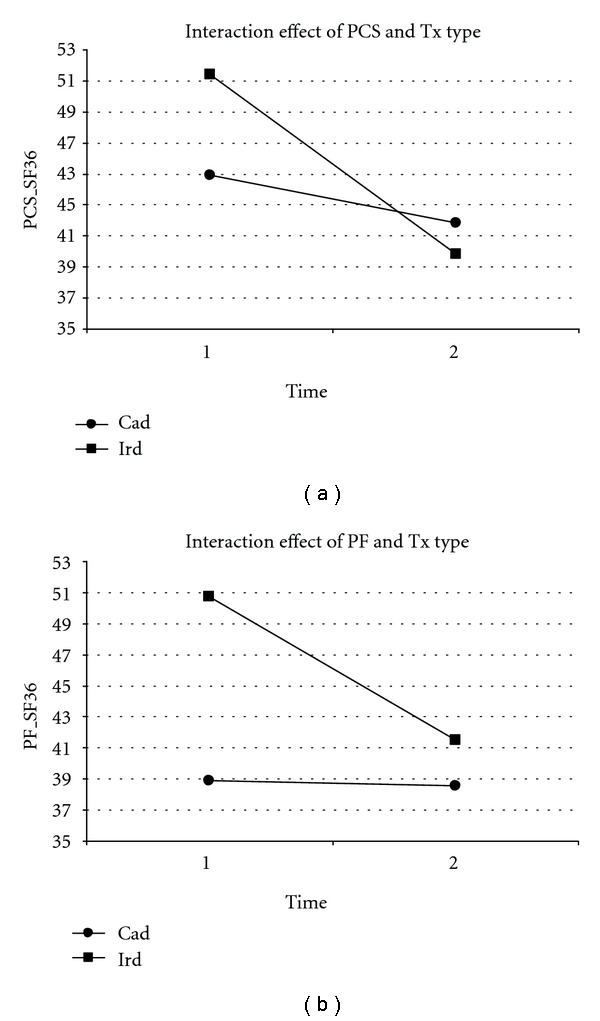


Post hoc tests showed that LRD transplant patients had higher physical functioning score and physical functioning scores at baseline compared to cadaver transplant (*F* = 5.72, *P* = .019, and *F* = 8.13, *P* = .005, resp.) but followup scores were equivalent. Within-groups post hoc tests showed that physical functioning declined significantly from baseline to 6-year followup in the LRD transplant group (*F* = 9.34, *P* = .007) whereas scores for cadaver transplant recipients remained unchanged (see Figure 2(a)). 

Physical component scores diminished in both transplant groups, yet the decrease was greater in LRD transplant patients (see Figure 2(b)).

A significant transplant type by time interaction effect was also found for disclosure (*F* = 4.817, *P* = .031). Post hocs tests revealed no group difference in absolute value between cadaver and LRD transplant recipients; yet LRD transplant recipients reported a nearing-significant increase in disclosure behaviours over time (*F* = 4.20, *P* = .055) whereas levels of disclosure remained unchanged in cadaver transplant patients (*F* = .003, *P* = .959).

### 3.4. Factors Associated with Tx-Specific and Generic HQoL Outcomes

#### 3.4.1. Factors Associated with Tx-Specific Outcomes

Correlation analysis between sociodemographic, medical variables and TxEQ sub-scales (absolute scores at followup) showed that increasing age was associated with less worry regarding the transplant (*r* = −.24, *P* = .023), more disclosure (*r* = .25, *P* = .019) and more perceived responsibility to do well (*r* = .22, *P* = .036) at the 6-year followup. 

Feelings of worry about the graft at followup were significantly higher in patients who perceived themselves as able of full or part time employment (mean = 3.33, SD =  .70) compared to patients who identified themselves as unable to work (mean = 2.95, SD =  .80) (*F* = 3.97, *P* = .049). 

Caucasian patients reported higher levels of disclosure about their transplant (mean = 4.08, SD =  .95) than patients of other ethnic groups (mean = 3.55, SD = 1.17) (*F* = 4.47, *P* = .037).

Only transplant vintage and perceived work ability were significantly associated with TXEQ change scores. Longer transplant vintage was associated with greater increase in feelings of guilt (*r* = .28, *P* = .008) and in feelings of responsibility across time (*r* = .27, *P* = .011).

Increase in worry about the transplant was greater in patients who perceived themselves to be capable of part or full time work (mean worry change score = 2.09, SD = 5.14) relative those who reported being unable to work (mean worry change score = −2.71, SD = 7.31) (*F* = 8.73, *P* = .004).

Significant associations were also found between the TXEQ sub-scales (absolute and change scores). Stronger feelings of guilt were significantly correlated with more worry about the transplant (*r* = .45, *P* = .001), higher perceived responsibility (*r* = .43, *P* = .001), and lower disclosure (*r* = −.43, *P* = .001). Feelings of responsibility to do well were positively associated with worry about the transplant (*r* = .35, *P* = .001) and adherence levels (*r* = .28, *P* = .006).

Guilt change scores were positively correlated with concomitant changes in worry (*r* = .39, *P* = .001) and responsibility (*r* = .30; *P* = .004), and negatively correlated with changes in disclosure (*r* − .32, *P* = .002). Worry about transplant change scores were also correlated with change scores in responsibility (*r* = .27, *P* = .015).

#### 3.4.2. Factors Associated with HQoL at Followup

No significant associations were found between HQoL and gender, ethnicity or measures of illness severity (haemoglobin levels, duration of dialysis prior to transplantation, time since transplantation) (data not shown). However, there were significant associations between number of comorbidities/long-standing illnesses, age, work status, perceived work ability, income, relationship status, Karnofsky scores and physical HQoL at followup in the expected direction (see Table 3). GFR was significantly associated with mental component score.

Significant associations were also found between worry about transplant and lower emotional HQoL at followup: mental component score, social functioning, mental health, vitality, and general health perception with correlation coefficients ranging from *r* = −.30 to *r* = −.27 (*P* ≤ .014). Disclosure shares a positive correlation with role-emotional (*r* = .24, *P* = .024), adherence to medication with Mental Component Score (*r* = .25, *P* = .029). Feelings of responsibility were negatively associated with physical component score, physical functioning and social functioning (see Table 3).

Univariate analyses on HQoL and TXEQ change scores revealed several significant associations: increased worry about viability of graft was associated with reduction in physical component score (*r* = − .32, *P* = .008), role physical (*r* = −.35, *P* = .004), role-emotional (*r* = − .32, *P* = .004), social functioning (*r* = −.45, *P* = .000), mental health (*r* = −.31, *P* = .006), bodily pain (*r* = −.24,   *P* = .028), vitality (*r* = −.297, *P* = .008), general health perception (*r* = −.47, *P* = .000), indicative of worse HQoL across time. Increase in guilt is related to role-emotional reduction (*r* = −.24, *P* = .028), and increase in adherence to medication is associated to a reduction in physical functioning (*r* = −.23, *P* = .040). 

HQoL changes were associated with income, perceived work ability, and relationship status in that HQoL losses were greater in the disadvantaged socioeconomic groups: low income and role-physical and social functioning (*F* = 4.11, *P* = .046; *F* = 7.03, *P* = .010, resp.); not able to work and role-emotional (*F* = 4.63; *P* = .036), social functioning (*F* = 8.47, *P* = .005) and mental component score (*F* = 7.32, *P* = .004), not in marital or cohabiting relationship (i.e., widowed, single or divorced/separated) and social functioning (*F* = 6.76, *P* = .011) and bodily pain (*F* = 5.20, *P* = .025).

### 3.5. Predictors of Generic Hqol Outcomes

Hierarchical multiple regression analyses were conducted to identify predictors of HQoL at 6-year followup. The variables selected for these analyses were those associated with physical component score and mental component score (absolute and/or change scores) in the univariate analyses at *P* < .05. Baseline levels of HQoL were entered first followed by sociodemographic clinical and psychological variables. Analyses were subsequently repeated using PCS and MCS change scores as the dependent variables.

The results indicated that baseline physical component score (beta =  .353, *P* = .014), worry about transplant (beta = −.30, *P* = .025), and perceived ability to work (beta = −.294, *P* = .039) were significant predictors of absolute physical component score at followup accounting for *R* ^2^ = 36% (Adj*R* ^2^ 31.1%) of variance. When variables were regressed on physical component score change scores the only significant predictor was worry change scores (beta = −.384, *P* = .012) explaining *R* ^2^ = 14.7% (Adj*R* ^2^ = 12.6%) of changes in physical HQoL over time. 

The regression model to predict mental component change scores in the combined transplant sample indicated that only perceived ability to work was significant (beta = −.442, *P* = .003), accounting for *R* ^2^ = 19.5% (Adj*R* ^2^ =17.5%) in mental component changes scores. Perceived work ability (beta = −.299, *P* = .026) and worry about transplant (beta = −.265, *P* = .043) were significant in the regression to predict absolute MCS at the followup jointly explaining *R* ^2^ =15.9% (Adj*R* ^2^ = 12.7%) of the variance.

## 4. Discussion

It has been long established that HQoL of transplant recipients improves from before to after receipt of the TX graft [1, 36, 37]. Previous studies have suggested that organ TX patients may reach a plateau at 1-2 years post-transplant with anticipated downturns thereafter but empirical evidence is lacking [19]. This study provides unique longitudinal data on the patient reported outcomes in kidney transplantation extending time frame of the analyses beyond the first 2 years posttransplantation. 

Study findings showed that the widely documented HQoL improvements pre- to post-transplantation [1, 38] cannot only be sustained but in some domains improved further over a much longer period. Our data indicate that decline in HQoL is not an inevitable consequence of long-term condition or advancing illness in the context of kidney transplantation—there is a small shift towards worsening of overall physical HQoL and domains most affected by health such as pain and physical functioning yet emotional dimensions of HQoL and vitality show marked improvements over time. 

Mean HQoL levels for combined sample and for both TX groups remained within 1 SD of general population norms at the followup yet individual HQoL trajectories were variable. Analysis of individual patterns showed differences in trajectories of change with 50% and 25% of transplant recipients reporting substantial decline of at least 5 points from baseline levels in physical component score and mental component score, respectively, indicating that group analysis may mask considerable interindividual variation in long-term outcomes. 

Although it may be tempting to attribute the observed changes to normal aging, studies of HQoL in cohorts of nonmedical community populations show HQoL across domains of physical and psychological health as well as emotional and social role functioning do not deteriorate substantially with aging but remain stable [39, 40]. Age was also not significantly associated with HQoL changes in this study. The deterioration in physical functioning, and role limitations levels over the 6-year window may be related to the chronic immunosuppressed state and the physical- and health-related consequences of long-term immunosuppression [41–43]. Physical HQoL impairments may also be related to incidence of diabetes which in renal transplant recipients as opposed to other transplant recipients is an end-organ/morbidity imposing disease rather an additional complication [44]. As such it may limit posttransplantation improvement or lead to physical HQoL reduction in renal recipients. As the number of diabetic patients in our sample was very small, the question of differential outcomes trajectories between patients with and without diabetes could not be examined.

The decline in physical dimensions functioning was evident in both transplant groups, yet the slope of deterioration was markedly greater for the LRD transplant recipients. The pronounced reduction of physical well-being in LRD transplant group is clearly noteworthy as clinical outcomes for these patients tend to be superior to those of cadaveric transplantation [45, 46]. Previous cross-sectional studies have reported comparable HQoL among transplant groups [7, 47], yet our longitudinal data suggest that physical HQoL is better preserved in CAD transplantation compared to LRD transplantation. Our data showed that physical HQoL in LRD transplant recipients is significantly higher than that of cadaver counterparts at baseline but diminishes dramatically over time to reach equivalent levels of those cadaver transplant patients at 6-year followup assessment. It is not clear what may be driving this differential pattern of changes over time in the two transplant groups. 

The findings albeit limited due to small numbers of LRD transplant patients suggest that a major area of improvement in transplant care is to preserve the physical HQoL benefits derived early on for this patient group. Although routinely the clinical care focuses mainly on adjustment of immunosuppression, prevention of complications, greater emphasis is needed on functional outcomes and rehabilitation throughout post-transplant care and beyond the immediate or early posttransplantation period. 

Another important finding is that despite physical quality of life declines, emotional well-being/ HQoL improved over time. Mean scores in mental health for instance, increased by nearly 1 SD from baseline to 6-year followup. These results contrast with findings of increased rates in anxiety and depressive disorders in kidney transplant recipients [48, 49] although the HQoL data was not designed to diagnose emotional disorders or measure specific emotions.

Despite the emotional HQoL gains, study findings indicate that patients may still have emotional needs/concerns. Worry about viability of the graft remained prominent in transplant recipients and increased significantly over time in line with the worsening physical HQoL evaluations. 

Late kidney graft loss remains a significant problem and hence patients increased feelings of worry about graft viability may reflect their awareness of the reality of finite graft function [50, 51]. Although clinical events or illness severity were not related to increased worry in this study it still remains likely that worry may be fuelled either through patients' direct experience of complications, bouts of ill health (not necessary related to renal condition) or vicariously when being informed of graft loss or death of other transplant patients. As shown in other settings [52] these indirect experiences can have a profound effect on surviving patients which may explain the increasing level of worry on one hand and improved emotional well being as patients may attach more significance and appreciate more their prolonged graft survival in the face on unpredictability of post-transplantation course.

The concomitant increase in worry about transplant viability and perceptions of responsibility to do well may suggest patients' resolve/determination to exert some control over graft uncertainty and unpredictability of prognosis or likely recurrence of underlying renal disease. It may be that perceptions of responsibility are acting as approach directed coping to maintain emotional morale and try to live up to expectations while putting fears and uncertainty behind them/aside.

It is also of note that worry about transplant was associated with both absolute and HQoL change scores, albeit, effect sizes were small. Patients whose HQoL declined also reported more worry about transplant. Attention should be particularly directed towards monitoring these concerns amongst the younger transplant recipients who appear to be more preoccupied/troubled with such concerns. Older patients reported lower levels of worry about transplant at followup despite worsening physical HQoL [53]. Interventions based on psychotherapy principles have been shown to be effective in addressing emotional issues in transplant recipients [9, 54].

The important question on predictors of HQoL changes in the kidney transplant population remains largely unanswered. We took into consideration several potential explanations for these findings but overall variance explained was fairly small—importantly, a number of illness and socioemotional variables albeit significantly associated with absolute HQoL scores consistent with previous cross sectional work [7, 42, 53, 55] were not consistently or reliably associated with the change in physical component score and mental component scores in the multivariate analysis. This clearly highlights the need for more longitudinal research to identify what drives changes in HQoL in this population. 

It is also important to recognize that there may other plausible explanations for the HQoL changes not explored in this study. Unmet expectations (discrepancy between health expectations and health experiences) are thought to adversely impact HQoL [56]. transplant patients may have high or even unrealistic expectations for life after kidney transplantation or even view their transplant as cure for their condition which may lead them to under rate physical HQoL and functional outcomes especially when complications or limitations are imposed or when rehabilitation or functionality related goals are not fully achieved. Further research is needed to examine patients' expectations on transplantation in the context of ESRD and to explore their role in determining immediate and long-term HQoL and emotional outcomes in kidney transplantation.

We acknowledge several limitations in this prospective observational study. First, even in longitudinal investigations issues of causality cannot be established with certainty as the findings remain associational in nature. In the absence of an experimental design, alternative explanations for the observed associations such as reverse causality and bidirectionality cannot be ruled out. Study participants were not assessed at a uniform post-transplant time although the time interval between assessments was fixed. However, to determine/control for the influence of years post-transplant, it was included as an independent variable in the analyses but shown no significant association with outcomes at followup. It is important to note that, as it was not deemed possible to conduct repeated sequential assessments, future prospective research would benefit from further methodological improvements such as including planned regular assessments of general and transplantation-specific outcomes at fixed time points throughout the early and long-term post-transplantation period.

The overall attrition rate may have resulted in a biased sample. This is partly due to the high turnover of transplant patients; with *N* = 38 patients being transferred out of our transplant unit and *N* = 36 not been traced at the 6-year of followup. Part of attrition was due to poor health with *N* = 21 patients refusing participation citing frail health, mobility issues or being overwhelmed with unresolved health issues which may have led to underestimation of magnitude of quality of life effects over the posttransplant period. 

Related to this, the sample size for the LRD transplant group was fairly small which may have undermined study power to detect significant differences between LRD and Cadaver renal transplant recipients. The study exclusion criteria of the present study may limit the generalizability of the results to the kidney transplantation population at large (i.e., individuals with psychosis, acute illness, neurological disease and other major organ, failure were not included in this study). 

Finally, caution is also warranted in interpreting study findings as *P* values were not adjusted for multiple comparisons, thereby resulting in inflated Type I error rate. The strategy adopted in this article was to be exploratory and to capitalize on chance findings to have greater certainty that no significant effects were overlooked [57]. Our discussion focuses mainly on significant findings (*P* ≤ .01). This highlights the need for further research to attempt to replicate or refute these findings.

## Figures and Tables

**Table 1:** Sociodemographic and clinical characteristics of study sample.

	Total sample (*N* = 102)	Cadaver transplant (*N* = 81)	LRD transplant (*N* = 21)	*P* value
	*M* (SD)/% (*N*)	*M* (SD)/% (*N*)	*M* (SD)/% (*N*)	
Age (years)	46.57 (14.21)	48.22 (14.67)	40.43 (10.57)	.025
Age (years) at diagnosis	31.18 (16.99)	32.81 (17.82)	25.04 (11.85)	ns
Education (years)	12.13 (4.46)	11.87 (4.80)	13.04 (2.94)	ns
Gender (% female)	41.2% (42)	39.5% (32)	47.6% (10)	ns
Ethnicity (% white)	76.5% (78)	75.3% (61)	81.0% (17)	ns
% married/cohabitating relationship	62.7% (64)	60.5% (49)	71.4% (15)	ns
% employed (f/t; p/t)	64.7% (66)	60.5% (49)	81.0% (17)	ns
% able to work	76.5% (78)	74.1% (60)	85.7% (18)	ns

Income				.012
*£*0–10,000	16.7% (17)	18.5% (15)	9.5% (2)	
*£*10,001–20,000	24.5% (25)	28.4% (23)	9.5% (2)	
*£*20,001–30,000	15.7% (16)	13.6% (11)	23.8% (5)	
>*£*30,000	21.6% (22)	14.8% (12)	47.6% (10)	ns

% Diabetes	6% (6)	6.1% (5)	4.7% (1)	ns
% Vascular disease	21.5% (22)	22.5% (18)	19% (4)	ns
% Hypertension	67.6% (69)	67.9% (55)	66.6% (14)	ns
GFR	43.83 (20.47)	43 (20.22)	45.77 (22.14)	ns
Transplant vintage (years)	8.08 (6.88)	11.87 (4.80)	11.25 (5.90)	.017

Primary kidney disease diagnosis (% yes)				
% GN	14.7% (17)	17.2% (14)	14.3% (3)	
% APKD	11.8% (12)	12.3% (10)	9.5% (2)	
% Reflux Nephropathy	8.8% (9)	9.8% (8)	4.76 % (1)	
% Diabetes	4.9% (5)	6.2% (5)	0% (0)	
% hypertension	13.7% (14)	16% (13)	4.6% (1)	
% All Other	44.1% (45)	38.3% (31)	66.6% (14)	

RRT: renal replacement therapies; f/t: full time; p/t: part time; ESRD-SI: end-stage renal disease severity index; GFR: glomerular filtration rate; GN: glomerulonephritis; APKD: adult polycystic kidney disease.

^†^ *P* values indicate significance of difference between CAD and LRD samples in the respective sociodemographic variables. ANOVA or Chi-square tests were used as appropriate.

**Table 2:** Patient reported outcomes at baseline and 6-year followup: total transplant sample; Cadaver transplant and living related transplant recipients.

	CAD TX		LRD TX		Total Sample		*P* value
	T1	T2	T1	T2	T1	T2	
	Mean (SD)	Mean (SD)	Mean (SD)	Mean (SD)	Mean (SD)	Mean (SD)	
*SF-36*							
MCS	45.84 (6.72)	50.82 (11.05)	43.50 (4.69)	49.73 (8.35)	45.30 (6.36)	50.57 (10.45)	.000
PCS	44.93 (12.30)	41.84 (12.29)	51.40 (7.15)	39.80 (14.70)	46.41 (11.61)	41.37 (12.80)	.001
PF	38.89 (16.91)	38.55 (16.47)	50.73 (6.21)	41.46 (13.79)	41.28 (16.06)	39.14 (15.94)	ns
RPh	45.88 (9.53)	45.06 (10.37)	51.14 (4.68)	46.73 (8.83)	46.94 (9.00)	45.39 (10.05)	ns
Rem	50.50 (6.88)	48.36 (10.01)	52.60 (4.17)	50.56 (7.10)	50.93 (6.45)	48.81 (9.49)	.037
SF	40.14 (10.38)	39.62 (14.65)	45.90 (7.27)	45.62 (11.24)	41.34 (10.06)	40.87 (14.17)	ns
MH	41.65 (9.19)	49.47 (12.59)	42.44 (5.67)	51.52 (9.36)	41.84 (8.54)	49.91 (11.96)	.000
VT	44.65 (7.18)	48.97 (10.92)	43.83 (6.09)	45.86 (10.24)	44.47 (6.94)	48.30 (10.80)	.000
BP	48.11 (11.40)	45.14 (13.15)	52.89 (8.29)	44.70 (13.91)	49.11 (10.95)	45.05 (13.24)	.002
GH	43.01 (11.55)	41.29 (13.14)	43.51 (10.04)	39.54 (9.72)	43.11 (11.21)	40.94 (12.51)	ns

*TxEQ*							
Worry	3.03 (.88)	3.25 (.71)	3.07 (.87)	3.30 (.77)	3.04 (.87)	3.26 (.72)	.036
Guilt	2.13 (.60)	2.25 (.78)	2.61 (.74)	2.55 (.73)	2.23 (.66)	2.32 (.77)	ns
Disclos	3.95 (.82)	3.96 (.99)	3.59 (1.15)	4.05 (1.04)	3.88 (.90)	3.98 (.99)	ns
Adherence	4.30 (.71)	4.28 (.76)	4.19 (1.11)	4.00 (1.00)	4.27 (.81)	4.22 (.82)	ns
Responsibility	3.72 (.71)	3.97 (.85)	3.71 (.74)	3.96 (.76)	3.72 (.71)	3.97 (.83)	.008

CAD: cadaver; TX: transplant; LRD: living related donor; T1: baseline; T2: 6-year followup; GH: general health; PF: physical functioning; BP: bodily pain; VT: vitality; RPh: role limitations due to physical problems; REm: role limitations due to emotional problems; SF: social functioning; MH: mental health; MCS: mental component score; PCS: physical component scores; TXEQ: Transplant effects questionnaire; Disclos: disclosure.

^†^Normative based scoring: In all SF-36 sub-scales the general population mean is 50 and 10 is a standard deviation.

^††^ *P* value reported based on repeated measure ANOVAs conducted on total sample.

**Table 3:** Correlations between HQOL at followup (absolute scores) and sociodemographic, clinical, and psychological variables.

	PCS	MCS	PF	RPh	Rem	SF	MH	VT	BP	GH
	*r* (*P* value)	*r* (*P* value)	*r* (*P* value)	*r* (*P* value)	*r* (*P* value)	*r* (*P* value)	*r* (*P* value)	*r* (*P* value)	*r* (*P* value)	*r* (*P* value)
Age	−.312 (.007)	ns	−.468 (.000)	−.294 (.005)	ns	ns	ns	ns	ns	ns
Education (years)	ns	ns	ns	ns	ns	ns	ns	ns	ns	ns
Corrected GFR	ns	.434 (.038)	ns	ns	ns	ns	ns	ns	ns	Ns
Number of Comorbidities	−.408 (.000)	ns	−.424 (.000)	ns	ns	ns	ns	−.301 (.003)	−.220 (.031)	−.291 (.004)
Number of long-standing illness	−.422 (.000)	ns	−.439 (.000)	ns	ns	ns	ns	−.315 (.002)	ns	−.279 (.006)
Transplant vintage (years)	ns	ns	ns	ns	ns	ns	ns	ns	ns	ns

*TXEQ subscales*										
Worry	ns	−.288 (.014)	ns	ns	ns	−.270 (.009)	−.299 (.003)	−.266 (.011)	ns	−.275 (.008)
Guilt	ns	ns	ns	ns	ns	ns	ns	ns	ns	ns
Disclosure	ns	ns	ns	ns	.243 (.024)	ns	ns	ns	ns	ns
Adherence	ns	.254 (.029)	ns	ns	ns	ns	ns	ns	ns	ns
Responsibility	−.312 (.007)	ns	−.376 (.000)	ns	ns	−.217 (.036)	ns	ns	ns	Ns

TX: Transplant; GH: general health; PF: physical functioning; BP: bodily pain; VT: vitality; RPh: role limitations due to physical problems; REm: role limitations due to emotional problems; SF: social functioning; MH: mental health; MCS: mental component score; PCS: physical component scores; TXEQ: transplant effects questionnaire; Ns: nonsignificant correlations.
